# Prevalence of COPD in other long-term conditions: a systematic meta review

**DOI:** 10.1186/s12890-026-04302-2

**Published:** 2026-04-22

**Authors:** Saleh N Al Sharmah, Rachel L. Clifford, Naiara T. Leonardi, Renata G. Mendes, Swapna Mandal, John R. Hurst

**Affiliations:** 1 UCL Respiratory, University College London, London, UK; 2 Centre for Respiratory Research, NIHR Nottingham Biomedical Research Centre, School of Medicine, Biodiscovery institute, University of Nottingham, Nottingham, UK; 3 Federal University of Sao Carlos, Sao Carlos, Brazil

**Keywords:** COPD, Prevalence, Long-term conditions, comorbidity

## Abstract

**Background:**

Chronic obstructive pulmonary disease (COPD) is a leading global health burden that often coexists with other long-term conditions (LTCs). However, the variable prevalence of COPD across LTCs is still under-recognised, which impacts the ability to implement integrated care strategies and targeted case-finding.

**Objectives:**

The aim of this meta-review was to summarise evidence from systematic reviews on the prevalence of COPD in the most prevalent LTCs.

**Methods:**

A systematic search of OVID, EMBASE, CINAHL and PubMed was conducted without date restrictions. Only systematic reviews published in English that reported COPD prevalence in LTC populations were included. The ROBIS tool was used to assess the risk of bias. The data extraction tool included author, year of publication, LTC classification, methods used to diagnose COPD, sample size, study settings, shared risk factors, prevalence estimates and odds/risk ratios.

**Results:**

Seventeen systematic reviews met the inclusion criteria. The prevalence of COPD varied across LTCs, ranging from 1.8% in axial spondylarthritis to 40.1% among lung cancer screening populations. Differences in COPD prevalence are likely to be influenced by variation in the LTC populations studied, the study settings and the methods used to identify COPD.

**Conclusion:**

COPD is prevalent across LTCs, which emphasises the need for multimorbidity care. The findings indicate that opportunistic COPD case-finding could be of benefit in settings for other LTCs. Standardisation of diagnostic criteria would make the comparability of studies more straightforward.

## Introduction

Chronic obstructive pulmonary disease (COPD) is a major global health burden and the third leading cause of death [1]. According to the 2019 Global Burden of Diseases (GBD) study, it accounted for approximately 3.23 million deaths [2]. This increased to 3.72 million deaths in 2021 [3]. COPD also imposes a substantial burden of lost life years and disability-adjusted life years (DALYs). Despite increasing recognition of COPD and its adverse impacts on individuals, the disease remains a challenge for global healthcare systems with underdiagnosis and undertreatment being common. One significant challenge is the prevalence of undiagnosed COPD. The international Burden of Obstructive Lung Disease study estimated that over 70% of people identified with COPD through spirometry had no previous diagnosis of chronic respiratory disease [4]. This under-recognition prevents timely interventions and increases the rate of adverse outcomes.

COPD is a condition that often co-exists with other long-term conditions (LTCs). Many people with COPD present with one or more comorbidities commonly including hypertension and cardiovascular diseases (CVDs) [5]. These coexisting conditions complicate the management and worsen clinical outcomes. Some of these conditions co-occur because of shared risk factors such as advancing age, or exposure to tobacco smoke and/or air pollution. Targeted detection of COPD in those with other LTCs may result in clinical benefits. Earlier COPD diagnosis may support early cessation of smoking, immunisation, pulmonary rehabilitation and pharmacological interventions, all of which can reduce disease burden and potentially impact outcomes of co-morbidities. In addition, detection of COPD in the context of other LTCs enables the creation of a more holistic care plan. Evidence indicates that case finding in those with LTCs could result in fewer hospitalizations and better management of health care resources [6]. 

The diagnosis of COPD is typically based on persistent respiratory symptoms together with airflow limitation confirmed by spirometry, usually defined as a post-bronchodilator FEV₁/FVC ratio of less than 0.70 [7] or the lower limit of normal. However, both in clinical practice and research, many different methods have been used to identify COPD including spirometry, self-report data, and electronic medical record codes [8]. Each of these methods has limitations. Access to spirometry is often limited [9], while self-report may fail to capture undiagnosed individuals or introduce misclassification [10]. Coding may or may not be accurate. Variation in methods used to diagnose COPD likely contributes to variation in COPD prevalence estimates across studies.

A number of individual systematic reviews have been published investigating the association between COPD and single LTCs indicating an urgent need to summarize all available research. A meta-review is an effective and structured way to collect and summarize findings from multiple systematic reviews. This strategy identifies common patterns of disease, gaps in evidence and supports the development of clinical guidelines and COPD case-finding strategies for populations with other LTCs. This meta-review aims to provide evidence by descriptively mapping existing systematic reviews that reported the prevalence of COPD in people with LTCs.

## Methods

### Search strategy

The meta-review was registered on PROSPERO (CRD42025644024). To decide which LTCs were most relevant we reviewed major health organizations’ disease classifications and reports, including the US Centers for Diseases Control and Prevention (CDC), Global Burden of Disease (GBD), United Kingdom Department of Health and Social Care, and World Health Organization (WHO). This process yielded a list of the most prevalent LTCs. HIV was included as a long-term condition of global health importance.

LTCs were grouped into categories according to commonly used disease classifications: cardiovascular diseases, mental health disorders, neurological disorders, musculoskeletal disorders, HIV and cancer.

A systematic literature search was conducted in four databases: OVID (Medline), EMBASE, CINAHL, and PubMed. The search was designed to identify systematic reviews that report the prevalence of COPD among individuals with the selected LTCs. Reviews were included if they conducted a systematic search strategy, focused on adults with one or more of the selected LTCs, and reported prevalence estimates of COPD in the LTC population. Only reviews published in English were included and there was no limit on date of publication. Reviews were excluded if they did not report data on COPD prevalence, were focused solely on treatment, management, prognosis, or disease mechanisms, or did not fit the definition of a systematic review. Narrative reviews, editorials, conference abstracts and case reports were excluded.

Data were extracted on the study settings and data sources reported within the included systematic reviews, where available. These were categorised as population-based, clinical (hospital- or clinic-based, including screening populations), administrative/database-based, or mixed. This was done to understand how methodological approaches varied, particularly the study populations and methods used to diagnose COPD.

### Search terms

The same search structure was applied across all databases, with adaptations made to suit database-specific indexing terms and syntax. Searches combined terms for COPD with terms for each selected LTC separately and prevalence-related terms. Controlled vocabulary searching was used where available including MeSH terms for MEDLINE and CINAHL subject headings in combination with free-text terms. Searches were conducted for each long-term condition separately which were then combined with the COPD terms using Boolean operators (AND/OR).

### Selection of relevant studies

Once the search had been completed and duplications removed, selected reviews were uploaded to RAYYAN software. Initial title and abstract screening were completed independently by two reviewers (SA and NTL) to select reviews that needed full-text screening. Disagreement was reconciled by discussion with a third reviewer (JRH). Full-text review was completed on all the studies that were still eligible.

### Risk of bias assessment

We used the ROBIS tool (Risk of Bias in Systematic Reviews) to examine the risk of bias. ROBIS assesses the methodological quality and reliability of systematic reviews [11]. Risk of bias assessment was conducted by the first author (SA). Greater emphasis was given to lower-risk reviews when interpreting COPD prevalence, the primary outcome of this meta-review. Other results sections were reported descriptively.

### Data extraction

Data from each of the reviews was extracted by one reviewer using a standardized data extraction form and verified by a second reviewer. The information extracted included details such as the authors’ names, year of publication, title, type of study and the number of studies listed. Further information was extracted on how the studies diagnosed COPD. Data on the prevalence (and odds ratio of COPD, if reported) for each LTC were recorded, including the point estimate, and lower and upper confidence intervals. Reference lists of primary studies included within each systematic review were extracted to identify overlap of the included studies across the reviews.

### Characteristics of included studies

**Fig. 1:**
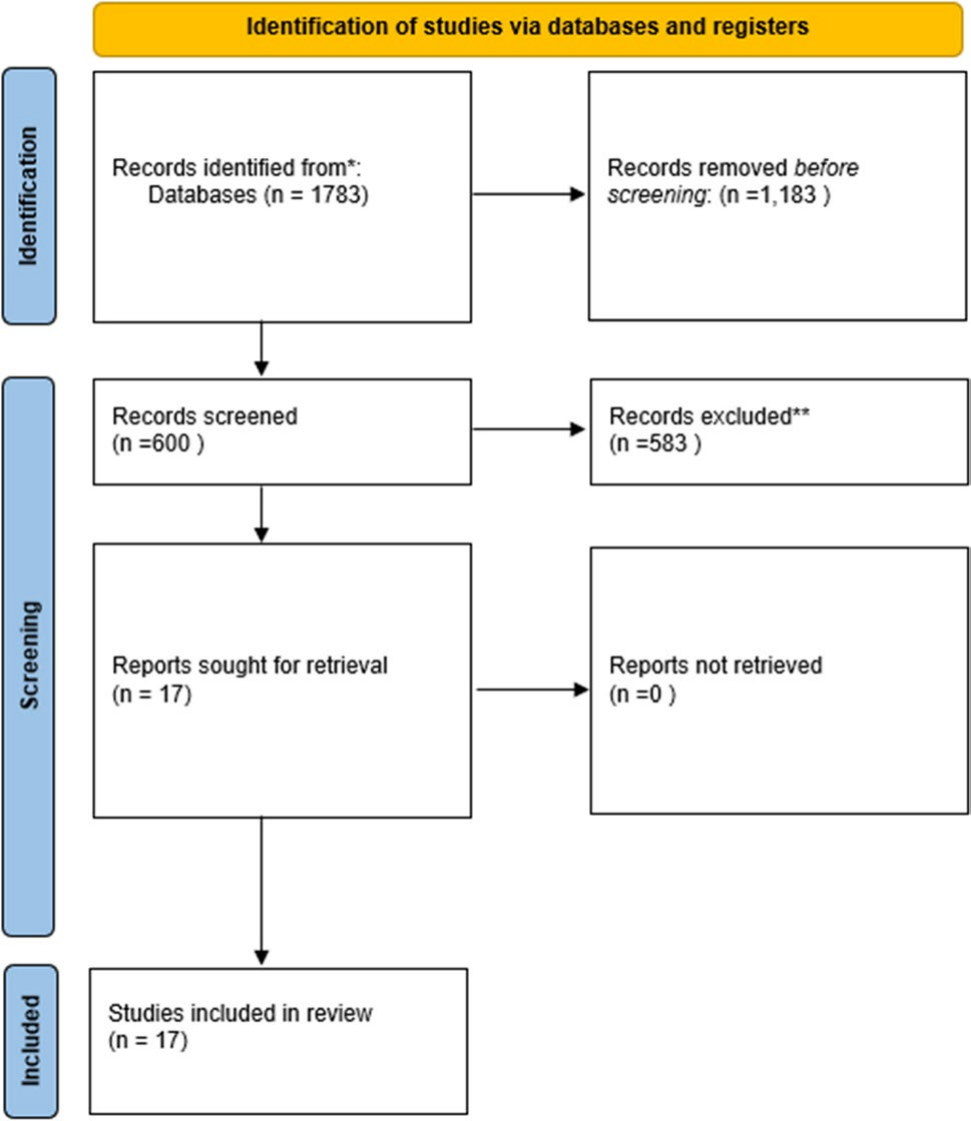
PRISMA flow diagram of study selections. A total of 1783 records were identified through four databases (OVID, EMBASE, CINAHL, PubMed). After removing 1.183 duplicates, 600 records were screened, of which 583 were excluded. 17 full-text reviews were assessed and all included in the final meta-review

A total of 1783 systematic reviews were imported from four databases. 1,183 were removed due to duplicates, leaving 600 reviews to be screened of which 583 were excluded (Fig. 1). In total, 17 systematic reviews were included in this meta-review. Examination of included primary study lists revealed no duplication of underlying studies across systematic reviews.

**Fig. 2:**
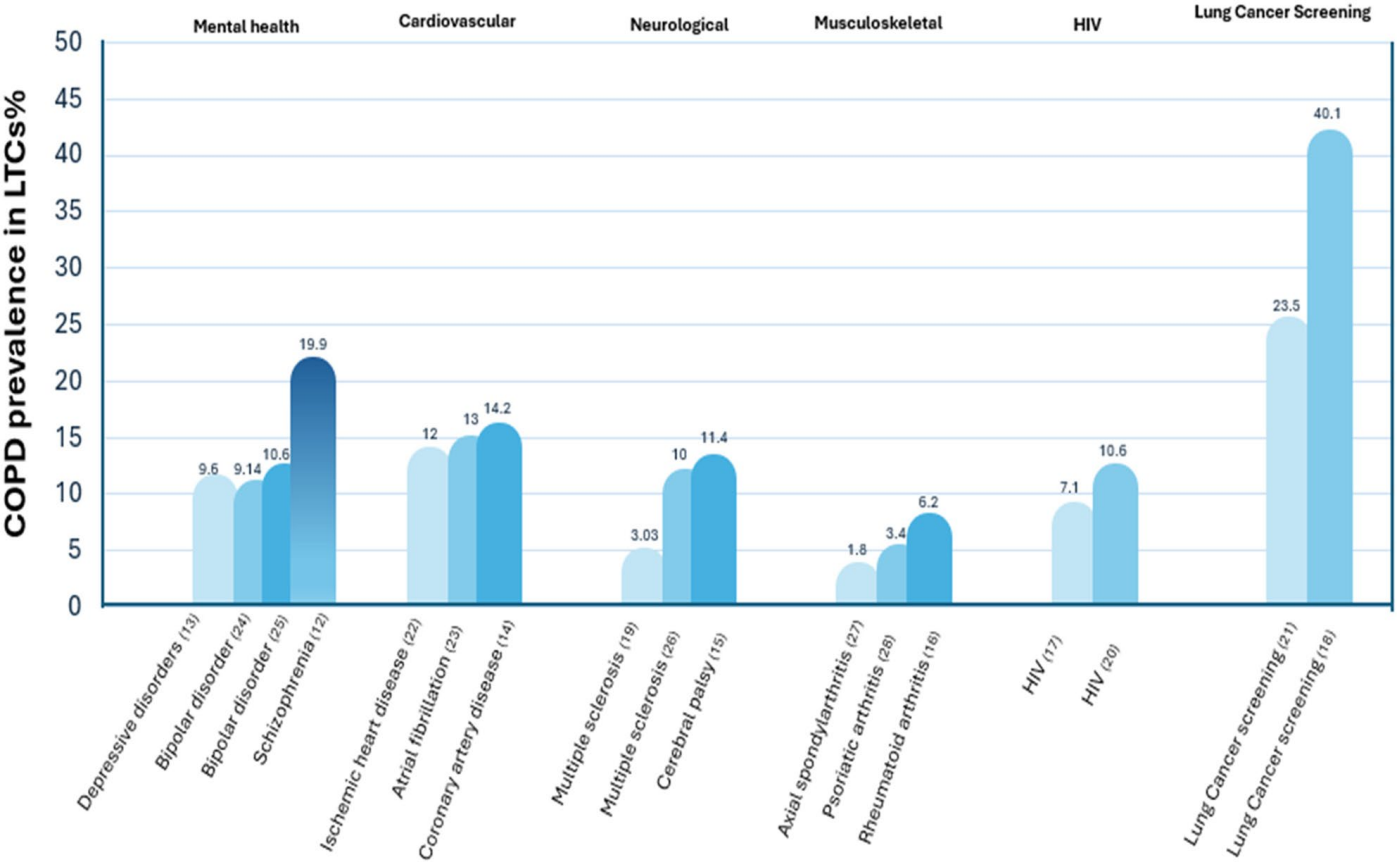
COPD prevalence across Long-Term Conditions. This figure summarises prevalence estimates of COPD reported in the included systematic reviews. The number of bars within each LTC group reflects the number of systematic reviews identified for that condition. Bars show the prevalence estimates reported in each review.

The results of the studies were reported in Table 1. Overall, we found a variable prevalence of COPD across LTCs (Fig. 2), with different approaches used in the diagnosis of COPD. Studies were of variable risk of bias (Table 1).

**Table 1:** Systematic reviews reporting the prevalence of COPD in other long-term conditions

	Authors/Year	LTC classification	Number of studies included	Sample size	COPD diagnosis	Study settings	Prevalence	Shared risk factors	Odd/Risk ratio	Risk of bias
1	(Jiménez-Peinado et al., 2025) [13]	Mental health	15 (14 COPD-related)	476,927 individuals with MDD assessed COPD, with additional analyses on asthma, pneumonia, and TB.	Medical diagnosis, medical records, or ICD criteria.	Community-based, hospital-based and mixed population.	Major depressive disorder (MDD) 9.0% (95% CI 3.8–19.6%), and it was slightly higher at 9.6% (95% CI: 3.0% to 27.2%) after bias adjustment.	Younger age associated with higher risk of developing COPD than the older age.	1.79 (95% CI: 1.49–2.15).	Low
2	(Laguna-Muñoz et al., 2025) [24]	Mental health	20 (15 COPD-related)	50,061 COPD individuals with BD.	Medical diagnosis, medical records, or ICD criteria.	Hospital, community and mixed settings.	Prevalence of COPD 9.14% (95%6.1–12.5%).	Younger age and higher proportion of women associated with increased COPD risk.	OR 1.73 (1.40–2.14).	High
3	(Montes et al., 2009) [25]	Mental health	21	Not reported .	Not reported.	Not reported.	10.6%	Not reported .	Not reported.	Low
4	(Suetani et al., 2021) [12]	Mental health	17 (14 COPD-related)	417,962 COPD individuals with schizophrenia.	Medical diagnosis, medical records, or ICD criteria.	Hospital, community and mixed settings.	in schizophrenia, COPD prevalence was 7.7% (95% CI: 4.0–14.4%), rising to 19.9% (95% CI: 9.6–36.7%) after adjusting for publication bias.	Older age associated with higher COPD prevalence.	OR 1.82, 95% (CI: 1.28–2.57).	Low
5	(Meng et al., 2024) [22]	Cardiovascular diseases	82	18 million individuals with ischaemic heart disease (IHD) evaluated for COPD across all the studies.	COPD was diagnosed by self-reporting regarding physician-diagnosis, and spirometry.	administrative databases, observational single-centre, observational multicentre, and RCT.	12% with a 95% (CI 9.9%-14.1%).	Age ≥ 70 years, smoking, diabetes, hypertension, CHF, CKD, and AF.	Hospital mortality OR 1.47 (95% CI 1.37–1.85); MACE 1.81 (1.44–2.27); AHF 2.14 (1.86–2.46); cardiogenic shock 1.30 (1.01–1.68); diabetes 1.17 (1.07–1.29); hypertension 1.23 (1.08–1.41).	High
6	(Romiti et al., 2021) [23]	Cardiovascular diseases	46	4,232,784 individuals with atrial fibrillation (AF).	The definition of COPD varied, with some studies using ICD codes including ICD-9 and ICD-10 versions and self-reported.	Secondary analyses of RCTs (*n* = 3), administrative databases (*n* = 19), multicentre observational studies (*n* = 16), and single-centre observational studies (*n* = 8).	13% (95% CI 10%-16%).	Diabetes mellitus, coronary artery disease, congestive heart failure, and stroke more common in patients with both AF and COPD.	All-cause death OR 2.22 (95% CI 1.93–2.55); hypertension 1.30 (0.97–1.73); diabetes 1.80 (1.38–2.35); CAD 1.84 (1.44–2.35); CHF 2.24 (1.73–2.90).	High
7	(Zheng et al., 2024) [14]	Cardiovascular diseases	65	6,400,000 individuals assessed for COPD.	PFT, ICD, or self-reported COPD.	Mixed clinical and administrative databases.	14.2% (95% CI: 13.3% to 15.1%)​.	Hypertension, diabetes mellitus, atrial fibrillation, stroke, smoking, dyspnoea, wheeze, and chronic bronchitis more frequent in COPD–CAD patients.	COPD group OR 1.94 (95% CI 1.57–2.40); hypertension 1.36 (1.20–1.53); diabetes 1.18 (1.10–1.27); stroke 1.72 (1.35–2.18); atrial fibrillation 1.64 (1.14–2.36).	low
8	(Ghoshouni et al., 2024) [19]	Neurological disorders	40 (19 COPD-related)	119,320 individuals with multiple sclerosis assessed for COPD.	Data sources included records, registries, questionnaires, ICD codes, physician diagnoses, and COPD medication history.	Clinical settings and population-based registry.	3.03% (95% CI: 1.82% to 5%).	Not reported.	OR 1.28 (95% 1.11–1.47).	High
9	(Marrie et al., 2015) [26]	Neurological disorders	249 (2 COPD-related)	Not reported .	Not reported.	Mixed (clinical, population-based and administrative databases).	10% with a (95% CI 0-20.9%).	Not reported.	Not reported.	Low
10	(Ryan et al., 2023) [15]	Neurological disorders	69 (7 COPD-related)	64,946 participants, with 7,723 identified with COPD.	Not reported.	Not reported.	11.4% (95% CI: 8.5%-14.7%).	Not reported.	Not reported.	Low
11	(Zhao et al., 2020) [27]	Musculoskeletal disorders	40 (8 COPD-related)	119,427 patients assessing COPD and other comorbidities.	Not reported.	Mixed (clinical, population-based and administrative databases).	1.8% with a (95% CI 0.9–2.8%).	Not reported.	Not reported.	High
12	(Gupta et al., 2021) [28]	Musculoskeletal disorders	39 (6 COPD-related)	158,797 patients with PsA, including 12,517 assessed for COPD.	Not reported.	Mixed (clinical and population-based).	3.4% (95% CI, 0-10.4%).	Not reported.	Not reported.	High
13	(Ma et al., 2019) [16]	Musculoskeletal disorders	14 (8 COPD-related)	102,205 patients with rheumatoid arthritis (RA) assessed for COPD.	Database codes and self-reported.	Mixed (database codes, population and clinical databased).	6.2% with (95% CI 4.1–8.3%).	Smoking.	RR 1.82 (95% CI 1.55–2.10).	Low
14	(Moyo-Chilufya et al., 2023) [17]	HIV	188 (5 COPD-related)	Not reported .	Not reported.	Clinical, community and database based.	CRDs prevalence (COPD and asthma) was 7.1% (95% CI: 4.0% – 10.3%).	Not reported .	Not reported.	Unclear
15	(Bigna et al., 2018) [20]	HIV	30	151 686 participants assessed to investigate the prevalence of COPD in HIV population.	ICD, Patient-reported, Post-bronchodilator (FEV₁) to (FVC) ratio less than 0.70 (fixed ratio), and post-bronchodilator FEV₁/FVC less than 5% of the age-dependent lower limit of normal (LLN).	Clinical and hospital based.	COPD prevalence was 10.5% (95% CI: 6.2–15.7%) by LLN and 10.6% (95% CI: 6.9–15.0%) by fixed-ratio definition.	Tobacco use, detectable HIV viral load, and country income level.	OR 1.14 (95% CI 1.05–1.25).	Low
16	(Almatrafi et al., 2023) [21]	Lung cancer	69 (11 COPD-related)	67,662 participants, including 9,868 individuals with COPD.	Based on medical records, GOLD and self-reporting.	Hospitals, primary care providers, healthcare systems, health insurance database, Lung screening trials.	23.5% with a 95% (CI 16.5%-31.4%), with advanced COPD (stages 3 and 4) is estimated at 10.7% (95% CI: 6.1, 16.4) in LCS.	Not reported.	Not reported.	High
17	(Choi et al., 2025) [18]	Lung cancer	42	126,842 individuals with emphysema and 72,209 with airflow limitation.	Spirometry-confirmed, LDCT imaging and self-reporting.	General population and clinical trials.	40.1% (95% CI, 33.4%-46.8%)​ in the LCS population.	Age > 60 years, smoking history > 40 pack-years, and male sex (> 60%).	Not reported.	Low

### COPD prevalence in long-term conditions

#### COPD prevalence in mental health

Four systematic reviews considered the prevalence of COPD in those living with mental health disorders, including depression, schizophrenia, and bipolar disorder. Schizophrenia, with a total of 417,962 individuals included in the assessment, was reported to have the highest prevalence of COPD among mental health disorders with an estimated prevalence of 19.9% (95% CI: 9.6–36.7%) [12] .Major depressive disorder showed a lower COPD prevalence at 9.6% ( 95% CI: 3.0-27.2%) [13].

#### COPD prevalence in cardiovascular diseases

Three systematic reviews examined the prevalence of COPD in individuals with cardiovascular diseases including atrial fibrillation (AF), ischemic heart disease (IHD) and coronary artery disease (CAD). The reported prevalence estimates varied by condition, with CAD showing the highest prevalence at 14.2% (95% CI: 13.3% to 15.1%), based on a pooled analysis of approximately 6.4 million participants, with prevalence highest in North America, followed by Asia, Europe, and South America [14].

#### COPD prevalence in neurological disorders

With regard to neurological disorders and disability, COPD prevalence was reported in three systematic reviews considering multiple sclerosis and cerebral palsy. The prevalence of COPD was higher in cerebral palsy compared to multiple sclerosis with a prevalence estimate of 11.4% (95% CI: 8.5%-14.7%) based on data from 7,732 participants [15].

#### COPD prevalence in musculoskeletal conditions

Rheumatoid arthritis was reported to have the highest prevalence estimates across musculoskeletal conditions with COPD prevalence reaching 6.2% (95% CI 4.1–8.3%), from data on 102,205 patients and giving an odds ratio of 1.82 [16]. COPD in this population was identified using database records and self-reported information. The prevalence of COPD among other musculoskeletal conditions such as psoriatic arthritis and axial spondylarthritis was lower.

#### COPD prevalence in HIV

Two systematic reviews reported COPD prevalence among HIV populations. The included studies used different methods to confirm the presence of COPD. One review focused on a population in sub-Saharan Africa, where the prevalence was 10.6% (95% CI: 6.9–15.0%) [17].

#### COPD prevalence in populations at risk of lung cancer

Two systematic reviews assessed the prevalence of COPD in cancer and/or cancer screening populations, particularly those undergoing lung cancer screening. COPD was diagnosed using spirometry and through self-report, with prevalence estimates that varied substantially reaching 40.1% (95% CI, 33.4%-46.8%) in a pooled analysis of 72,209 individuals [18].

### COPD association with long-term conditions

Some of the included reviews that reported COPD prevalence also reported data on the magnitude of the association between COPD and these conditions. Increased odds ratios were identified in mental health disorders including major depressive disorder (OR 1.97 (95% CI: 1.49–2.15)_ and schizophrenia (OR 1.82 (95% CI: 1.28–2.57)) [12, 13]. A significantly higher odds ratio for COPD was also seen in those with cardiovascular diseases, particularly coronary artery diseases (OR 1.94 (95% CI: 1.57–2.40)) [14]. Among musculoskeletal conditions, the risk ratio (RR) for COPD was approximately doubled in rheumatoid arthritis (RR 1.82 (95% CI: 1.55–2.10)) [16].

### Sample size and study population

The included systematic reviews included data from a range of populations and clinical settings. Sample sizes ranged from 50,000 in mental health disorders to 18 million participants in ischaemic heart disease populations. A majority of studies were conducted in secondary care settings whereas a smaller number focused on community-based or screening populations. Cardiovascular diseases, mental health and musculoskeletal disorders had the largest cohorts, while neurological disorders, HIV and lung cancer populations involved smaller populations.

### COPD diagnostic methods

Diagnostic approaches for COPD were heterogeneous among the included studies. In mental health disorders, COPD was mainly diagnosed based on medical records or ICD codes with limited use of spirometry. In cardiovascular diseases, the diagnosis of COPD was generally based on a combination of spirometry, ICD codes and self- or physician-reported diagnosis. The diagnostic method impacted the reported COPD prevalence, particularly in CAD patients, as subgroup analysis showed that using spirometry yielded higher prevalence estimates (21.3%) compared to ICD codes (14.6%) or self-reported data (8.8%) [14]. For neurological disorders, such as multiple sclerosis, the definition of COPD relied on medical records, registries, self-reports, ICD codes and physician diagnosis [19]. In musculoskeletal diseases, specifically rheumatoid arthritis (RA), COPD identification was limited to database codes and self-reported diagnosis, while in HIV, ICD coding, patient-reported COPD, and spirometry were utilised to diagnose COPD with, once again, prevalence reported to be lower when relying on ICD or patient-reported diagnosis compared to spirometry [20]. Finally, within lung cancer screening populations, COPD was diagnosed using medical records, self-report, or spirometry, and the prevalence estimates varied with health records higher at 38.7% compared with those using self-reporting at 16.9% [21].

### Shared risk factors

Across the included reviews, several risk factors between COPD and other LTCs were noted. Smoking was the most consistent reported risk factor in cardiovascular diseases, rheumatoid arthritis, mental health disorders and HIV. In cardiovascular diseases, COPD was related to age ≥ 70 years, smoking, hypertension, diabetes mellitus, congestive heart failure, chronic kidney disease, atrial fibrillation, stroke and chronic bronchitis [22, 23, 14]. In mental health disorders, the findings were mixed, younger age and female sex were associated with increased risk in bipolar disorder and depressive disorder [13, 24], while older age was associated with COPD among people with schizophrenia [12]. Additionally, among HIV populations, tobacco use, detectable viral load and country income were cited as factors associated with increased COPD risk [20]. Lastly, older age (> 60 years), heavy smoking history (> 40 pack-years), and male sex (> 60%) were reported as key risk factors for COPD among lung cancer screening population [18].

## Discussion

A total of 17 individual systematic reviews have examined the prevalence of COPD across a range of LTCs, including mental health disorders, cardiovascular diseases, HIV, musculoskeletal conditions and cancers. The prevalence of COPD across LTC populations varied, with the highest prevalence reported in lung cancer screening populations, and lowest prevalence in musculoskeletal disorders.

The presence of COPD in patients with other physical LTCs has been associated with poor COPD outcomes, increasing the rates of exacerbations and lowering quality of life [29], linked with increasing disease severity, symptom burden and adverse health outcomes [30]. Furthermore, COPD is associated with psychological distress, including anxiety and depression, which can negatively affect treatment adherence and thus impact disease progression [31].

Despite the high prevalence and significant impact, under-diagnosis of COPD remains common. Individuals with airflow limitation may remain undiagnosed for a number of reasons, including because respiratory symptoms can be misattributed to another condition [32]. This makes the detection of COPD more complex in the presence of other LTCs such as cardiovascular diseases. Studies have reported that 70% to 80% of patients with COPD identified through case-finding have not been previously diagnosed [6]. This gap contributes to poorer health outcomes and higher healthcare utilisation.

Shared risk factors contribute to the high prevalence of COPD in people with coexisting LTCs, such as tobacco smoke exposure and ageing. The higher prevalence of COPD in rheumatoid arthritis may also be explained by such shared risk factors but also systemic inflammation, with smoking linked to the production of anti-citrullinated protein antibodies (ACPA) which associate with RA [33]. Importantly, our results suggest missed opportunities to identify co-existing COPD in the context of care for other LTCs during management of the primary condition. Such approaches have been tested, for example in the context of hypertension, both in high- [34] and lower income settings [35].

Heterogeneity in diagnostic methods likely influenced the reported prevalence. The approach to COPD diagnosis was inconsistent across studies. Some studies used spirometry, the gold standard, generally finding a higher prevalence while other studies used medical records or self-reported data to identify COPD and were generally associated with a lower prevalence. The included papers covered populations in different regions with different healthcare systems and environmental exposure which could also have contributed to variation in prevalence. This variation limited the extent to which prevalence estimates can guide targeted COPD case-finding strategies in LTC populations.

We found that COPD is prevalent across LTCs which supports the need for integrated, multimorbidity care strategies. COPD can often remain undiagnosed in individuals being treated for other LTCs, especially CVDs, HIV, and mental health disorders. Routine care for these groups should incorporate opportunistic case-finding tools to enable early detection and management of COPD to prevent complications which may lead to a negative impact on LTC outcomes [36]. Improving the care for LTCs such as heart failure and diabetes has been associated with better COPD outcomes [37, 38]. This demands an approach to multi-disciplinary, holistic care and emphasises the importance of supporting primary care where much care for LTCs is delivered.

In non-respiratory clinics, case-finding may be particularly useful when patients present with clinical indicators of COPD, including breathlessness, chronic cough, sputum or wheezing, especially where there is a history of smoking or other relevant exposures [39]. Embedding spirometry in non-respiratory clinics could help detect undiagnosed COPD [40]. Spirometry in primary care helped to identify a substantial number of patients with undiagnosed COPD, with newly detected rates ranging from 4.1% to 40.2% [41]. Similar findings have been observed in atrial fibrillation clinics [42]. Overall, the higher prevalence estimates observed when spirometry is used likely reflect the high presence of undiagnosed COPD in these populations.

Enhancing clinician and patient awareness and education is critical to address underdiagnosis of COPD. Many clinicians managing LTCs outside respiratory clinics lack confidence in conducting or interpreting spirometry [43]. Targeted education and inter-professional training can build clinicians’ capacity to effectively identify and manage patients with COPD [44]. Similarly, improving patient awareness of respiratory symptoms is essential, as some patients may be in denial or may prioritize other health issues over respiratory symptoms [45]. Supporting smoking cessation is critical [46]. Implementing these strategies can support earlier detection and prevention of COPD across healthcare settings.

This review has strengths. A comprehensive search strategy was conducted across four major databases including: OVID, EMBASE, CINAHL, and PubMed. We used predefined inclusion criteria focusing on the most prevalent LTCs with the greatest burden. As a meta-review, we only included systematic reviews to ensure high-level evidence. Lastly, the use of ROBIS assessed the risk of bias in the included systematic reviews.

Despite these strengths, there were limitations. The inconsistency in the diagnosis of COPD across studies affects the comparability of prevalence estimates. Limiting the search to English-language publications may have introduced bias. Additionally, most reviews did not group results by key variables such as age, sex or smoking status which limited the ability to identify subgroups at highest risk. We note that there were no reviews published to study COPD prevalence for some important conditions including hypertension, diabetes, chronic kidney disease and liver disease. Whilst some studies reported odds ratio, most reviews did not include a control population to understand the magnitude of any increased risk.

## Conclusion

We summarize evidence on the burden and co-existence of COPD among populations living with a range of LTCs. We demonstrate the importance of approaching COPD as a component of multiple long-term conditions. Despite methodological differences across studies, the consistency of findings across a broad range of LTCs suggests the co-existence of COPD and LTCs is a common and under-recognized area. Future research should direct efforts to address and overcome the limitations observed in this review, in particular insufficient data on underrepresented yet prevalent conditions such as hypertension, diabetes, chronic kidney disease and liver disease. There is also more data required on strategies evaluating case-finding for COPD in the context of other LTCs. In conclusion, evidence supports identification and integrated management of COPD in individuals with LTCs, as an important opportunity to improve outcomes, and support more holistic, patient-centred care.

## Data Availability

All data analysed during this study are derived from previously published studies and are cited within the manuscript.

## Appendix

The search strategy was developed to identify systematic reviews reporting the prevalence of chronic obstructive pulmonary disease (COPD) among individuals with selected long-term conditions (LTCs). The same search structure was applied across all databases, with adaptations made to suit database-specific indexing terms and syntax.

Searches combined terms for COPD with terms for each selected long-term condition and prevalence-related terms. Controlled vocabulary searching was used where available, including MeSH terms for MEDLINE and CINAHL subject headings, in combination with free-text terms. Searches were conducted for each long-term condition separately and then combined with COPD terms using Boolean operators (AND/OR). A filter for systematic reviews and meta-analyses was applied. Risk factor terms were not included as they were beyond the scope of this review.

### Search keywords:

- **COPD terms:** Chronic obstructive lung disease, chronic bronchitis, emphysema
- **Prevalence terms:** Prevalence, prevalence rate, existence, occurrence, coexistence
- **CVD terms: **cardiovascular disease, CVD, heart illness, coronary heart illness, ischemic heart illness, heart failure, myocardial infarction,
- **Ischemic heart disease terms:** coronary heart disease, coronary artery disease, CHD, CAD, myocardial infarction, heart attack
- **Mental health terms:** mental illness, psychiatric disorder, depression, schizophrenia, bipolar disorder, psychosis, anxiety
- **Musculoskeletal disease terms:** Musculoskeletal diseases, arthritis, osteoarthritis, rheumatoid arthritis, osteoporosis, back pain, joint disease, muscle disease,
- **Neurological disorder/ Dementia terms:** neurological disorders, neurologic diseases, neurodegenerative diseases, epilepsy, seizure disorders, Parkinson's disease, Alzheimer's disease, multiple sclerosis, cerebrovascular disease, cognitive impairment, neurocognitive disorder, vascular dementia
- **Cancer terms:** neoplasm, malignancy, tumor, carcinoma, oncology
- **HIV terms:** HIV, Human immunodeficiency virus, HIV infection, HIV-1, HIV-2, Acquired immunodeficiency syndrome, AIDS, HIV/AIDS, HIV positive
- **Hypertension terms:** Hypertension, High blood pressure, Elevated blood pressure, Essential hypertension, Primary hypertension, Secondary hypertension, Arterial hypertension, Systemic hypertension
- **Chronic Kidney Disease terms:** chronic kidney disease, chronic renal disease, chronic kidney failure, chronic renal failure, chronic kidney insufficiency, chronic renal insufficiency, CKD, kidney dysfunction, renal dysfunction
- **Diabetes terms:** diabetes mellitus, diabetes, type 2 diabetes, T2DM, type II diabetes, diabetes mellitus type 2
- **Liver disease terms:** chronic liver disease, liver cirrhosis, hepatitis, hepatitis B, hepatitis C, liver fibrosis, liver failure, hepatic disease
- **Stroke terms:** Stroke, cerebrovascular accident, brain infarction, cerebral infarction, ischemic stroke, hemorrhagic stroke, transient ischemic attack

## Abbreviations

COPD: Chronic Obstructive Pulmonary Disease
LTC: Long-Term Condition
CVD: Cardiovascular Disease
CKD: Chronic Kidney Disease
HIV: Human Immunodeficiency Virus
